# Hormetic Effect Caused by Sublethal Doses of Glyphosate on *Toona ciliata* M. Roem

**DOI:** 10.3390/plants12244163

**Published:** 2023-12-15

**Authors:** Giselle Santos de Faria, Leandro Carlos, Adriano Jakelaitis, Samylla Tassia Ferreira de Freitas, Taíza Andressa Vicentini, Igor Olacir Fernandes Silva, Sebastião Carvalho Vasconcelos Filho, Lucas Loram Lourenço, Fernanda Santos Farnese, Marco Aurélio Batista, Luciana Cristina Vitorino

**Affiliations:** 1 Programa de Pós-Graduação em Ciências Agrárias, Instituto Federal de Educação, Ciência e Tecnologia Goiano (IF Goiano, Campus Rio Verde), Rodovia Sul Goiana, Km 01, Zona Rural, Rio Verde 75901-970, GO, Brazil; giselle.punk@hotmail.com (G.S.d.F.); leandro.carlos@ifgoiano.ediu.br (L.C.); adriano.jakelaits@ifgoiano.edu.br (A.J.); samyllatassia@hotmail.com (S.T.F.d.F.); igorolacirv95@gmail.com (I.O.F.S.); 2 Programa de Pós-Graduação em Biodiversidade e Conservação, Instituto Federal de Educação, Ciência e Tecnologia Goiano (IF Goiano, Campus Rio Verde), Rodovia Sul Goiana, Km 01, Zona Rural, Rio Verde 75901-970, GO, Brazil; vicentinitaiza@gmail.com (T.A.V.); sebastiao.vasconcelos@ifgoiano.edu.br (S.C.V.F.); lucas.loram@outlook.com (L.L.L.); fernanda.farnese@ifgoiano.edu.br (F.S.F.); 3 Programa de Pós-Graduação em Recursos Naturais do Cerrado, Universidade Estadual de Goiás, BR-153, Km 99, Qd. Área, Km 99, Campus Bairro São João, Anápolis 75132-903, GO, Brazil; m_batista@outlook.com.br

**Keywords:** dose response, hormetic effect, herbicides, physiological toxicity, timber species

## Abstract

This study aimed to evaluate the response of *Toona ciliata* seedlings to sublethal doses of glyphosate. The increasing use of glyphosate in agriculture concerns the scientific community, as the drift of this pollutant into aquatic systems or atmospheric currents can affect non-target species. Therefore, we need to understand how non-target species respond to small doses of this herbicide. *T. ciliata* seedlings (clone BV-1110) were exposed to sublethal doses of glyphosate (0, 9.6, 19.2, 38.4, 76.8 g ae ha^−1^). Anatomical, physiological, and photochemical analyses were performed 60 days after herbicide application, and growth assessments were carried out after 160 days of cultivation. We found that sublethal doses of glyphosate above 19.2 g ae ha^−1^ induced toxicity symptoms in *Toona ciliata* leaves. These symptoms were mild in some cases, such as chlorosis, but severe in other cases, such as tissue necrosis. We observed a positive relationship between increased plant height and photochemical yield with plant exposure to sub-doses 9.6 and 19.2 g ae ha^−1^. A sublethal dose of 38.4 g ae ha^−1^ improved the photosynthetic rate and carboxylation efficiency. Thus, we confirmed the hypothesis of a hormetic effect when *T. ciliata* was exposed to sub-doses of glyphosate equal to or lower than 38.4 g ae ha^−1^. However, the sublethal dose of 76.8 g ae ha^−1^ must be considered toxic, impacting photosynthetic activity and, consequently, the height of *T. ciliata*. The stem diameter of *T. ciliata* responded positively to increasing glyphosate doses. This occurs to compensate for the negative effect of glyphosate on water absorption. Further research will provide valuable information for harnessing the potential benefits of hormesis to improve the productivity of *T. ciliata*.

## 1. Introduction

*Toona ciliata*, a tree species belonging to the family *Meliaceae*, is widely distributed in several countries across continents [1]. However, it is a wild plant nationally protected in China due to the threat of extinction resulting from intense deforestation and low natural propagation rates [1,2,3]. This species was introduced in Brazil, and as it has excellent adaptation to the climate, it is exploited for commercial wood production. In this country, there is an overestimation of forest plantations in terms of their contributions to timber production and environmental conservation [4]. Research focusing on the factors affecting endangered species is directed towards the conservation and cultivation of important species, such as *T. ciliata*, which is widely recognized for its excellent red heartwood quality and rapid wood growth rate [2,5,6]. Despite their importance, *T. ciliata* populations are pressured not only by deforestation and fragmentation events but also by physiological damage caused by the drift of herbicides commonly used in adjacent agricultural cultivation areas.

Glyphosate is a non-selective post-emergent herbicide used worldwide [7]. Its mechanism of action involves inhibition of the EPSPs enzyme (5-enolpyruvylshikimate-3-phosphate synthase), resulting in a broad spectrum of systemic actions against weeds [7,8,9,10]. Glyphosate acts on the shikimate pathway, inhibiting the synthesis of the essential aromatic amino acids phenylalanine, tyrosine, and tryptophan, which are precursors of other compounds, such as lignin, alkaloids, flavonoids, and benzoic acids [11,12]. Additionally, glyphosate is used as a plant growth regulator, maturation agent [13], and desiccant [14]. Its foliar absorption is rapid, compromising the synthesis of chlorophyll and carotenoids [7]. We know that the continuous use of pesticides in agriculture occurs with the aim of improving the quality of food products and reducing the number of plant diseases, but the occurrence of pesticides in wastewater and atmospheric currents worries the scientific community since these pollutants have harmful effects and are persistent, undergoing bioaccumulation resulting in risks to human health and environmental damage due to their toxicity [15,16]. In sensitive plants, glyphosate induces irreversible cellular damage that includes chloroplast rupture, cell plasmolysis, hyperplasia, cell proliferation, wax layer removal, and cuticle disruption, resulting in cell collapse and necrosis [17].

There is an imminent need to identify tree species with productive timber potential for cultivation in different regions of the world to promote diversity in forest production [18,19,20,21]. In this context, *T. ciliata* has great potential in tropical regions, where it finds favorable conditions for its vegetative development in a manner similar to that required by native cedars [22]. However, worldwide agricultural production is based on the frequent application of herbicides, which can affect areas of vegetation and the cultivation of adjacent wood species. Many species are negatively affected by lethal doses of herbicides; however, sublethal doses can positively affect growth and physiological activity [23,24,25]. According to Vilela et al. [26], the use of glyphosate is not recommended in *T. ciliata* cultivation areas, and mechanical control of weeds is necessary, but Cedergreen [27] demonstrated that the herbicide glyphosate stimulates growth in several plant species when applied at doses of 5–60 g ae ha^−1^, corresponding to realistic spray drift events. Thus, we decided to test the hypothesis that sublethal doses of glyphosate could affect the anatomy and physiology of *T. ciliata* plants, inducing hormesis or compromising structures and physiological and photochemical responses.

Anatomical and physiological analyses of plant specimens are important for understanding plant responses to herbicides [28,29]. Anatomical analyses allow for the observation and monitoring of structural changes in different plant structures, such as vascular tissues, epidermal cells, and leaf morphology, which can be affected by glyphosate [30]. Physiological analyses assess metabolic processes such as photosynthesis, respiration, and transpiration and provide insights into the overall health of the plant and its ability to respond to different doses of glyphosate. Thus, it is possible to observe whether herbicide doses influence the efficiency of photosynthesis, the transpiration rate, and other aspects that indicate the functional state of plants [31].

By combining anatomical, physiological, and data related to the primary photochemistry of chlorophyll *a*, we achieve a comprehensive understanding of the effects of glyphosate doses on plants, allowing inferences about underlying mechanisms. This is crucial for the accurate interpretation of results and for guiding future herbicidal usage decisions [32,33]. Promoting studies and conservation efforts are essential to preserve and tap into the potential of *T. ciliata*, utilizing sub-doses of glyphosate for growth stimulation through the hormesis effect [34,35]. However, by observing the imminent influence of these chemicals on plants, the use of plant species as bioindicators has emerged as an important alternative for assessing the potential impacts of this herbicide on biota and in biomonitoring of exposures [17].

## 2. Results

### 2.1. Visual Symptoms and Anatomical Assessments

**Figure 1:**
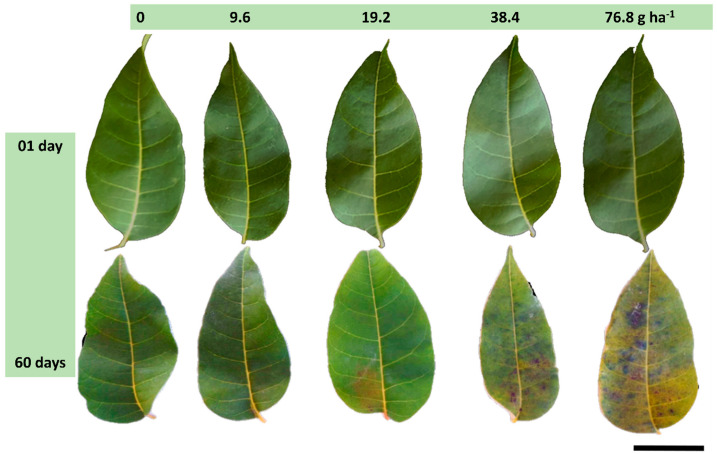
Visual morphological analysis on the leaves of *Toona ciliata*, on the 1st and 60th day after glyphosate application at different sublethal doses (0 g ha^−1^; 9.6 g ha^−1^; 19.2 g ha^−1^; 38.4 g ha^−1^; and 76.8 g ha^−1^). Scale bar: 3 cm.

No visual symptoms were observed in the leaf blades 24 h after exposure to sublethal doses of glyphosate, regardless of the applied dose. However, after 60 d, chlorosis followed by necrosis was observed, along with the proliferation of microorganisms within the darkened regions with whitish coloration, which became more noticeable at a dose of 76.8 g ha^−1^. Symptoms appeared at 19.2 g ha^−1^ and intensified at higher doses (Figure 1).

**Figure 2:**
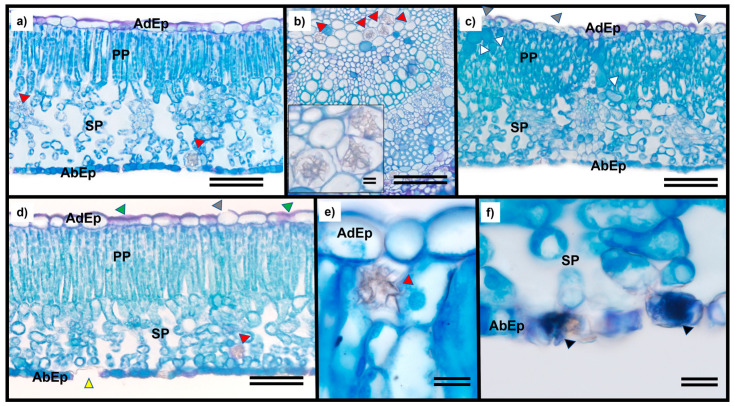
Leaf anatomy of *Toona ciliata* exposed to different sublethal doses of glyphosate: (**a**,**b**) 0.0, (**c**) 9.6, (**d**) 19.2, (**e**) 38.4, and (**f**) 76.8 g ha^−1^. (AdEp) Adaxial epidermis, (AbEp) Abaxial epidermis, (PP) Palisade parenchyma, and (SP) Spongy parenchyma. (**a**–**d**) Scale bar 100 µm, and (**e**,**f**) Scale bar 25 µm. Black arrows indicate necrosis; green arrows indicate plasmolysis; red arrows indicate crystal presence; white arrows indicate idioblasts; gray arrows indicate cuticle loss, and yellow arrows indicate loss of epidermal cells.

Anatomical analyses of *T. ciliata* leaves revealed cellular and structural damage on all doses of glyphosate. *T. ciliata* species exhibit leaf epidermis on the adaxial and abaxial surfaces of the leaves, composed of isodiametric cells with flat or slightly convex anticlinal walls, and are considered hypoestomática. The mesophyll was dorsiventral and consisted of one or two layers of palisade parenchyma, five to seven layers of spongy parenchyma cells, and idioblasts with calcium oxalate druses, similar to the control (Figure 2a). Druse-type crystals were observed in the primary veins and mesophyll regardless of the sublethal dose (Figure 2b). A sublethal dose of 9.6 g ha^−1^ caused the removal of the cuticular layer and alterations in the epidermis of both surfaces and in the mesophyll, leading to hyperplasia and cellular proliferation in the palisade parenchyma cells (Figure 2c).

Starting at a dose of 19.2 g ha^−1^, mucilage was observed in the epidermis of both surfaces, with the adaxial epidermis showing cuticle rupture and loss of continuity in the abaxial epidermis (Figure 2d). The presence of idioblasts with druses was observed at the dose of 38.4 g ha^−1^ (Figure 2e), whereas a dose of 76.8 g ha^−1^ caused necrosis of cells in the abaxial epidermis (Figure 2f).

### 2.2. Physiological Assessments

**Figure 3:**
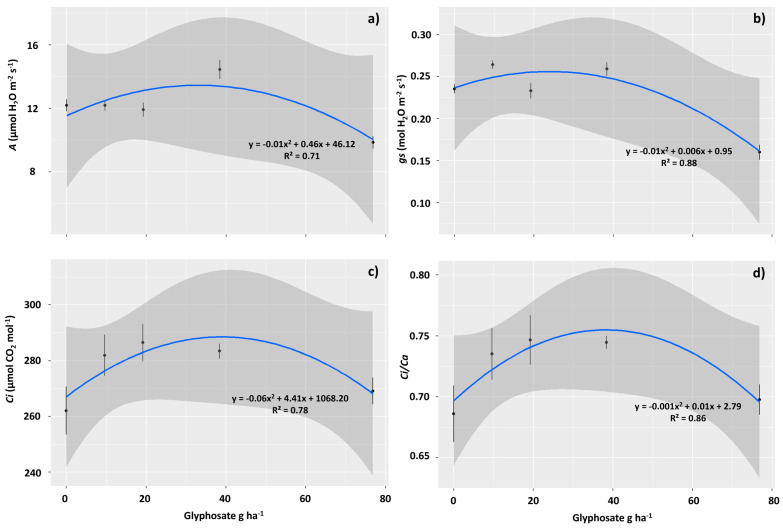
Gas exchanges observed in *Toona ciliata* plants exposed to different sublethal doses of glyphosate: 0.0, 9.6, 19.2, 38.4, and 76.8 g ha^−1^. Net carbon assimilation rate (*A*) (**a**), stomatal conductance (*gs*) (**b**), internal CO_2_ concentration (*Ci*) (**c**), and the ratio of internal to external CO_2_ concentration (*Ci*/*Ca*) (**d**). The straight lines represent the fitted model, and the prediction intervals (95%) are gray. The points represent the averages and the vertical lines the SE.

Physiological analyses revealed that net carbon assimilation (*A*) exhibited a quadratic pattern, with a maximum increase of up to 24% in the photosynthetic rate at a dose of 38.4 g ha^−1^ (Figure 3a). Similar behavior was observed for *gs*, with a maximum increase of 12% observed at a dose of 19.2 g ha^−1^ (Figure 3b). The internal CO_2_ concentration (*Ci*) also exhibited a quadratic pattern of behavior, depending on the increase in the sublethal dose of glyphosate, with an increase of 9% in plants subjected to 19.2 g ha^−1^ of the herbicide compared with the control (Figure 3c). The ratio of internal to external CO_2_ concentration (*Ci*/*Ca*) showed identical behavior to that observed for *Ci*, reaching the highest averages in plants exposed to 19.2 g ha^−1^ of glyphosate. An increase of 8.7% in this ratio was observed in glyphosate-free plants (Figure 3d).

**Figure 4:**
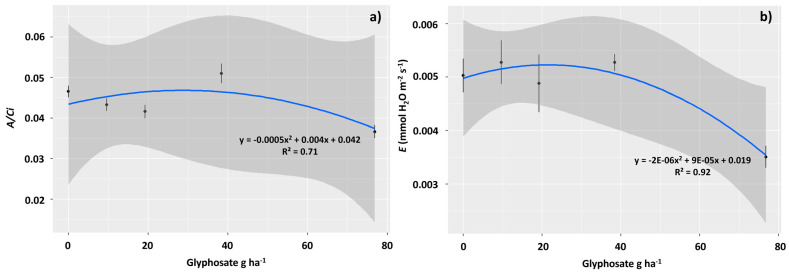
Gas exchanges observed in *Toona ciliata* plants exposed to different sublethal doses of glyphosate: 0.0, 9.6, 19.2, 38.4, and 76.8 g ha^−1^. Carboxylation efficiency (*A*/*Ci*) (**a**), and transpiration rate (*E*) (**b**). The straight lines represent the fitted model, and the prediction intervals (95%) are gray. The points represent the averages and the vertical lines the SE.

Carboxylation efficiency (*A*/*Ci*) was also significantly affected by exposure to glyphosate, with the highest efficiencies observed in plants exposed to 38.4 g ha^−1^, with a 10.8% increase in efficiency compared to the control plants (Figure 4a). The highest transpiration rates (*E*), however, were observed in plants treated with 9.6 and 76.8 g ha^−1^ of the herbicide, with an increase of 4% in relation to the control in plants sampled in these two treatments (Figure 4b).

**Figure 5:**
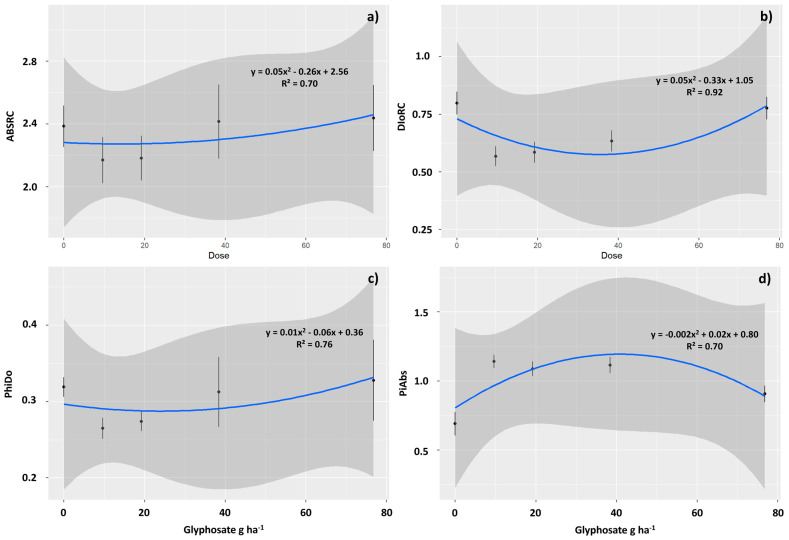
Chlorophyll *a* fluorescence observed in *Toona ciliata* plants exposed to different sublethal doses of glyphosate: 0.0, 9.6, 19.2, 38.4, and 76.8 g ha^−1^. Specific light absorption flux per reaction center (ABSRC) (**a**), specific dissipated energy flux at the level of the chlorophyll antenna complex (DioRC) (**b**), energy dissipation yield (PHIDo) (**c**), and photosynthetic performance index (PiAbs) (**d**). The straight lines represent the fitted model, and the prediction intervals (95%) are gray. The points represent the averages and the vertical lines the SE.

Exposure to glyphosate also affected the chlorophyll *a* fluorescence. Plants exposed to concentrations of 9.6 and 19.2 g ha^−1^ exhibited the lowest average values of specific light absorption flux per reaction center (ABSRC) (Figure 5a), indicating a reduction in photochemical damage. The ABSRC was reduced by 9.1 and 8.5% in these plants, respectively. Thus, the specific dissipated energy flux at the level of the chlorophyll antenna complex (DioRC) was also reduced in these plants, indicating less energy dissipation in the form of heat (Figure 5b). In these plants, DioRC was reduced by 28.8 and 26.7%, respectively, compared to the control plants. Energy dissipation yield (PHIDo) followed the same pattern and was reduced in plants subjected to these treatments. The reductions were respectively 17.2 and 14.1% in plants treated with 9.6 and 19.2 g ha^−1^ glyphosate, respectively (Figure 5c). Consequently, the photosynthetic performance index (PiAbs) was higher in plants subjected to these treatments as well as in those subjected to 38.4 g ha^−1^ of glyphosate. The averages were 65.2, 56.6 and 60.8% higher than the control in plants treated with 9.6, 19.2, and 38.4 g ha^−1^ of herbicide (Figure 5d).

### 2.3. Biometric Assessments

**Figure 6:**
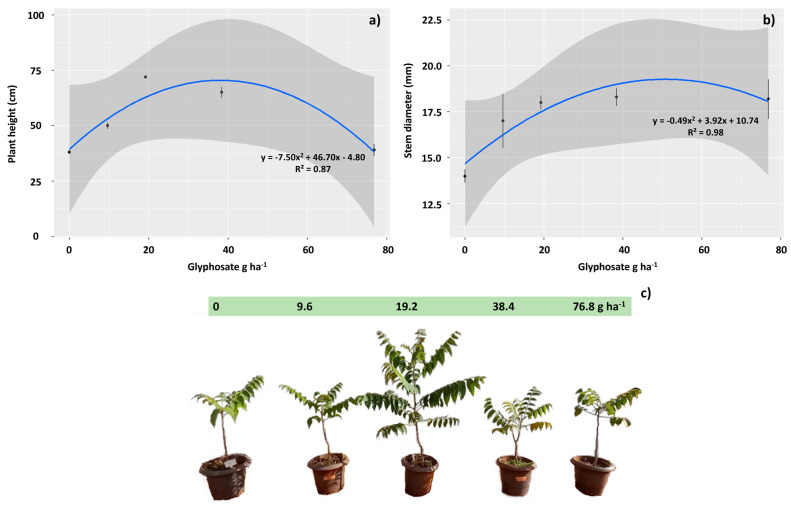
The average relative growth rate in *Toona ciliata* at 160 days after sublethal doses of glyphosate application: 0.0, 9.6, 19.2, 38.4, and 76.8 g ha^−1^. Height (**a**), stem diameter (**b**), and total development of plants (**c**). The straight lines represent the fitted model, and the prediction intervals (95%) are gray. The points represent the averages and the vertical lines the SE.

The relative growth after 160 days of herbicide application revealed that *T. ciliata* seedlings exposed to the herbicide benefited from the treatments of 19.2 and 38.4 g ha^−1^ of glyphosate, with the best dose response observed at 19.2 g ha^−1^. However, the doses of 9.6 and 76.8 g ha^−1^ showed a reduction in height growth rate over 160 days (Figure 6a,c). Stem diameter increased in all glyphosate-treated groups, with the best responses observed at 19.2, 38.4, and 76.8 g ha^−1^ (Figure 6b).

### 2.4. Principal Component Analysis and UPGMA

**Figure 7:**
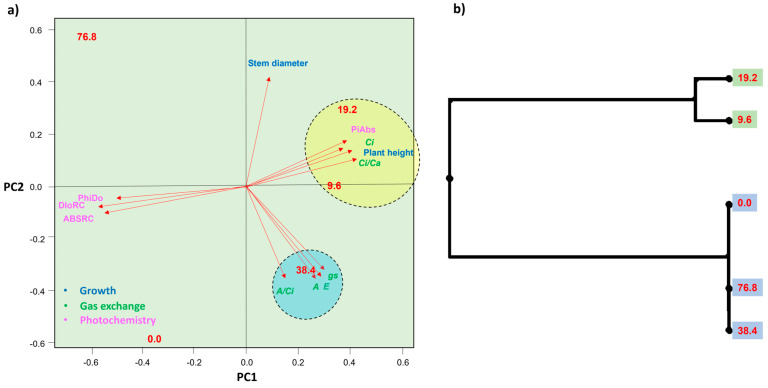
Principal component analysis recovered for *Toona ciliata* plants exposed to different sublethal doses of glyphosate: 0.0, 9.6, 19.2, 38.4, and 76.8 g ha^−1^ (**a**) and dendrogram of the mean distance (UPGMA) of physiological, photochemical and growth variables considered (**b**). Net carbon assimilation rate (*A*), stomatal conductance (*gs*), internal CO_2_ concentration (*Ci*), and the ratio of internal to external CO_2_ concentration (*Ci*/*Ca*), carboxylation efficiency (*A*/*Ci*), transpiration rate (*E*), specific light absorption flux per reaction center (ABSRC), specific dissipated energy flux at the level of the chlorophyll antenna complex (DioRC), energy dissipation yield (PHIDo), and photosynthetic performance index (PiAbs).

Principal components 1 and 2 together explained 98.3% of the data variance, with the highest average values for the variables of plant height growth, photochemical efficiency (PiAbs), *Ci* and the *Ci*/*Ca* ratio being positively related to plants grown under sublethal doses 9.6 and 19.2 g ha^−1^ of glyphosate. The best photosynthetic responses, however, involving *A*, *gs*, *E* and the *A*/*Ci* ratio, were positively related to plants treated with 38.4 g ha^−1^ herbicide (Figure 7a). UPGMA (unweighted pair-groups method using arithmetic averages) confirmed the similarity between the data obtained for plants exposed to 9.6 and 19.2 g ha^−1^ of glyphosate. These formed a cohesive cluster, distant from the plants exposed to 0.0, 38.4, and 76.8 g ha^−1^, which also grouped together based on the set of traits evaluated (Figure 7b).

## 3. Discussion

### 3.1. Toxicity Symptoms Were Observed in T. ciliata Leaves Applying Sublethal Doses of Glyphosate above 19.2 g ha^−1^

Changes were also observed in the cuticle, epidermis, and leaf mesophyll. The cuticle constitutes the main barrier leaves for the absorption of toxicants, and when this structure is compromised, glyphosate interferes with carbon metabolism and induces chlorophyll *a* fluorescence [36]. This explains the photosynthetic and photochemical damage observed in plants subjected to the highest sublethal dose, where epidermal necrosis was observed. Therefore, this dose was considered toxic to *T. ciliata*. Similarly, Cruz et al. [28] exposed *Eugenia uniflora* L. plants to glyphosate and observed alterations in the chloroplasts and phloem, the tissues responsible for plant transport. Glyphosate directly inhibits chlorophyll biosynthesis and can lead to necrotic regions, as observed in studies conducted by Oliveira et al. [37].

The epicuticular wax layer, which is mainly composed of a network of aliphatic compounds and is lipophilic in nature, according to Bianchi and Bianchi [38], provides protection to leaves. The removal of this structure by the application of glyphosate at doses as low as 19.2 g ha^−1^ renders the plant vulnerable. This can worsen at higher doses, such as 76.4 g ha^−1^, which can inhibit photosynthesis. Dos Reis et al. [39] stated that the cuticle contains several polar channels that serve as entry pathways for herbicides. Given the heterogeneity of the epicuticular wax layer on leaves, these transcuticular pathways are composed of carbohydrate polymers that extend from the cuticle to the cell wall.

However, visual assessment of foliar symptoms should not be used as the sole indicator, as the herbicide may not directly cause symptoms but could be due to other factors, such as plant susceptibility to pathogen attacks. This was observed in *T. ciliata* seedlings, which exhibited no visible symptoms after glyphosate exposure. However, anatomical analyses revealed the removal of the wax layer and cuticle rupture, and over time, the leaves became diseased without visible changes as a result of the applied dose. Treatments at 38.4 g ha^−1^ or higher resulted in removing the wax layer from the leaves, rendering the plants vulnerable to pathogen attack, which caused the analyses over 60 d to be more affected than expected. Yilmaz and Dane [40] reported that cuticular waxes can be absent or reduced in regions such as the veins and guard cells of stomata, which may be associated with the discontinuity of abaxial epidermal cells. Lower wax content and reduced hydrophobicity of the cuticle are related to increased leaf sensitivity and greater translocation through vascular bundles [41,42].

In general, foliar damage attracts microorganisms that compromise plant defense barriers. This is yet another abiotic stress that *T. ciliata* requires to allocate energy to combat it. However, this energy can also be used for vegetative growth. Energy depletion for defense purposes can be achieved through chlorophyll degradation, which affects vegetative growth, as observed in *Bowdichia virgilioides* leaves infested by fungi following glyphosate exposure [17]; cellular plasmolysis, hyperplasia, cell proliferation, and cellular collapse with necrosis symptoms were also observed in *B. virgilioides* and *Eucalyptus grandis* W. Hill ex Maiden clone leaves [17]; and in *C. brasiliense* leaves subjected to glyphosate drift simulation [43].

Necrosis was observed by Freitas-Silva et al. [44] in the mesophyll cells of *H. chrysotricus*. In this *T. ciliata* study, necrosis was observed in the abaxial epidermal cells. Oliveira et al. [37] identified cellular plasmolysis in *B. virgilioides* leaves, whereas Cruz et al. [28] observed plasmolysis and cellular collapse in *E. uniflora* leaves exposed to glyphosate. The presence of druse-type crystals in plants is related to plant adaptation and was found in the control samples as well as in the other treatments. These crystals are associated with defense against herbivory and toxicity [45], heavy metal detoxification [46], ion balance, and pollen tube development, and serve as a source for forming this structure. The growth of pollen tubes requires intracellular calcium gradients [47,48,49,50], which are more abundant near vascular bundles [51] and serve as calcium (Ca^2+)^ reserves in case of increased demand [52]. According to Cavalcante et al. [53], calcium oxalate is an endogenous source of carbon for photosynthetic reactions.

### 3.2. The Sublethal Dose of 76.8 g ha^−1^ of Glyphosate Negatively Affected the Photosynthetic Performance of T. ciliata

Associated with intense leaf damage, including tissue necrosis, the sub-dose of 76.8 g ha^−1^ affected the gas exchange of *T. ciliata*, which resulted in lower carbon assimilation in the plants. Therefore, although it constitutes a dose below that commonly used in agriculture to eliminate weeds, this dose triggered symptoms of toxicity in *T. ciliata*. The toxic effects of glyphosate on photosynthetic activity are associated with its indirect effects on chlorophyll molecules. Glyphosate acts systemically in plants and is transported via the phloem, resulting in the inhibition of the enzyme enol-pyruvyl shikimate phosphate synthase (EPSPS) and the inhibition of chlorophyll synthesis. Santos et al. [29] also observed leaf necrosis and a reduction in photosynthetic capacity in *Cenostigma macrophyllum* plants subjected to the application of this herbicide. These authors reinforce the idea that the reduction in *gs* and the consequent reduction in *E* and *A* indicate the action of the herbicide on guard cells, causing stomatal closure. However, the reduction in *A*/*Ci* indicated that this damage occurred not only in the stomata. There was probably a decrease in CO_2_ diffusion in the mesophyll, damage, or a decrease in the amount of the RuBisCO enzyme, which is responsible for CO_2_ fixation in the Calvin Cycle. Tissue necrosis may have contributed to the deficiency in CO_2_ diffusion in the mesophyll. A dose of 76.8 g ha^−1^ was able to decrease g, an indication of stress, which in turn is related to stomatal closure, limited growth, and plant production [54]. These effects draw attention to the possibility that plants present in vegetation fragments immersed in agricultural matrices are affected by the spread of glyphosate in cultivation areas [55].

Despite the toxic effect of 76.8 g ha^−1^, a lower concentration, that is, 38.4 g ha^−1^ of the herbicide, improved the photosynthetic yield of *T. ciliata*, and induced a hormetic effect. This indicates that low-dose exposure favors photosynthetic activity in plants. This effect is characterized by metabolic benefits induced by exposure to low concentrations of toxins [56]. Other studies have highlighted the possibility of sublethal doses of glyphosate-inducing effects in plants of different species. Nadeem et al. [57] demonstrated that the application of glyphosate in the range of 65–250 g ha^−1^ stimulated the germination and seedling growth of the weeds *Coronopus didymus*, *Chenopodium album*, *Rumex dentatus* and *Lathyrus aphaca*. *Cicer arietinum* L. plants had their growth and productivity increased by applying sub-doses of this herbicide [58]. According to Brito et al. [59], glyphosate at low rates can increase plant growth, induce the accumulation of shikimic acid, increase photosynthesis and stomatal opening, increase seed production, and shorten the life cycle of plants. This herbicide appears to play a prominent role in the shikimic acid pathway, reducing lignin synthesis and resulting in the improved growth and productivity of several crops [60]

### 3.3. Sublethal Doses of 9.6 and 19.2 g ha^−1^ of Glyphosate Improved Photochemical Performance in T. ciliata Leaves

According to Da Silva et al. [61], glyphosate interferes with the activity of the enzyme 5-enolpyruvylshikimate-3-phosphate synthase (EPSPS), responsible for the synthesis of several amino acids essential for plant development [62]; however, we found that plants treated with the lowest sublethal doses had better primary photochemical performance through hormesis, as they were low doses, without toxicity effects. PSII is the most sensitive component of the photosynthetic apparatus and plays an important role in photosynthetic responses to toxicological factors [63,64]. PSII uses light energy to oxidize water into molecular oxygen and provides electrons and protons, being more susceptible than photosystem I (PSI) to photodamage. The hormetic response of PSII is triggered by the non-photochemical fluorescence quenching (NPQ) mechanism, which is a strategy to protect the photosynthetic apparatus from photo-oxidative damage, dissipating excess light energy as heat and preventing the synthesis of destructive reactive oxygen species (ROS). Moustakas et al. [65] suggest that a basal level of ROS is required for optimal plant growth, whereas a low increase in ROS is beneficial for triggering hormetic responses, and a high level of ROS outside of the boundaries is considered harmful to plants. This explains the effects we observed when exposing *T. ciliata* plants to doses of 9.6 and 19.2 g ha^−1^ of glyphosate, which induced a better photochemical performance index and less energy dissipation in the form of heat, which indicates a reduction in stress caused by the incidence luminous. Similarly, Costa et al. [66] showed that glyphosate applied between doses of 4.4 to 55 g ha^−1^ in *Coffea arabica* plants improves the photochemical efficiency of PSII.

### 3.4. Sublethal Doses of 9.6, 19.2 and 38.4 g ha^−1^ of Glyphosate Stimulate the Growth of T. ciliata Plants

These effects were observed in terms of the plant height and stem diameter. According to Yamashita and Guimarães [67], sublethal doses of glyphosate can promote stem thickening in young plants of *Azadirachta indica*. Substress caused by the application of sub-doses of glyphosate promoted stem diameter thickening in *T. ciliata* at all tested doses. Low doses of glyphosate can induce growth in different plant species via hormesis [8,54]. It is suspected that an initial increase in plant growth when sprayed with low doses of herbicides could give these plants a head start, leading to a higher final biomass and corresponding seed yield [27].

Doses between 19.2 and 38.4 g ha^−1^ of glyphosate, although causing anatomical symptoms in the species, did not result in growth stagnation. In contrast, the species showed improved growth rates at 160 DDA compared to the control, exhibiting a hormetic effect [17,43]. Hormese stimulate chemicals can affect the plant morphology or metabolic processes [39]. Previous studies evaluating the hormetic effects of the herbicide glyphosate have shown biomass production and stem diameter stimulation at low doses [43,68,69]. The apical meristems of the stem, responsible for plant height growth, emitted new branches and, consequently, leaves, indicating that sub-doses of glyphosate were insufficient to interfere with auxin synthesis [8].

The reduction in A in plants grown under a sublethal dose of 76.8 g ha^−1^ resulted in lower rates of production or transport of assimilated photosynthates and, consequently, reduced height. According to Costa et al. [66], these alterations are responsible for growth decline. However, a dose of 76.4 g ha^−1^ induced stem thickening, similar to the dose used by Marques et al. [68], who applied 60 g ha^−1^ to *Cedrela odorata*, a species of the same family (Meliaceae), also known as cedar.

In this study, the herbicide doses were chosen considering that *T. ciliata* is sensitive to glyphosate, and based on previous greenhouse experiments conducted by the research group, we demonstrated that sublethal doses of 9.6, 19.2, or even 38.4 g ha^−1^ could positively stimulate photosynthetic and photochemical metabolism, as well as the growth of plants of this species. Thus, we verified the hormetic effect of the interaction between low doses of the herbicide glyphosate and *T. ciliata* plants. We drew attention to the fact that, in general, doses higher than 76.8 g ha^−1^ are used in agricultural spraying; thus, owing to drift, they can compromise the survival of *T. ciliata* plants found in vegetation adjacent to planting areas.

## 4. Materials and Methods

### 4.1. Experimental Design and Conduct

The experiment was conducted from November 2021 to July 2022 in the experimental area of Instituto Federal Goiano, Rio Verde Campus, Goiás, Brazil. Clonal seedlings of Australian cedar (*T. ciliata*) BV-1110 were obtained from the Bela Vista Nursery in Campo Belo, Minas Gerais, Brazil, and replanted in 5 L pots filled with non-cultivated soil in a 3:1 ratio. The fertilization followed the recommendation of Carlos et al. [70] using potassium at 0.128 kg ha^−1^, ammonium sulfate at 0.36 kg ha^−1^, and MAP (monoammonium phosphate) at 0.40 kg ha^−1^ dissolved in 5 L of water. Irrigation was calculated based on the soil analysis to meet the nutritional needs of the plants. The physicochemical characteristics of the soil (dystrophic Red Oxisol) were as follows: pH 5.12, 1.06 cmolc dm^−3^ Ca, 0.48 cmolc dm^−3^ Mg, 1.6 Ca + Mg, 0.05 cmolc dm^−3^ Al, 2.1 H + Al, 0.33 cmolc dm^−3^ K, 128 mg dm^−3^ K, 8.4 mg dm^−3^ S, 2.6 mg dm^−3^ P, and clay 48%, silt 12%, and sand 40%.

The plants were kept in a greenhouse for 30 days to acclimatize them to the experimental conditions. After this period, the plants were subjected to five different sublethal doses of glyphosate, with each treatment evaluated five times, totaling 25 sample units of *T. ciliata*. The sublethal doses applied were: 0, 9.6, 19.2, 38.4, and 76.8 g ae ha^−1^, with a single application, which verified the high sensitivity of *T. ciliata* to doses above 76.8 g ha^−1^ of glyphosate. The herbicide glyphosate (Roundup Transorb^®^, 480 g ha^−1^ of acid equivalent) was applied using a backpack sprayer with constant pressure maintained by compressed CO_2_. The sprayer was equipped with a four-nozzle bar and an XRTeejet^®^ flat fan nozzle (model XR11002—VP). A total volume of 120 L ha^−1^ was applied. The application occurred at 2:00 p.m., with a wind speed of 1.3 m s^−1^, average temperature of 28.2 °C, and relative humidity of 73.4%. Climatic conditions were monitored during cultivation, with temperatures varying between 27–30 °C and relative humidity between 80–57%.

### 4.2. Visible Leaf Symptoms

One day after glyphosate application, fully expanded *T. ciliata* leaves were photographed using a semi-professional camera (Cyber-Shot SONY HX100V) to record visible chlorosis and necrosis symptoms on the leaf surface. The same methodology was repeated 60 d later to compare the observed visual changes.

### 4.3. Leaf Morphoanatomical Characterization

To observe subvisual symptoms, morphoanatomical characterization of leaves exposed to different doses of glyphosate was performed. Leaf samples measuring 3 mm were collected 1 day after glyphosate treatment. Samples were collected from the margin, middle, and center regions of the last fully expanded leaf in all replicates (*n* = 5) from each treatment (*n* = 5) of *T. ciliata* plants. After collection, samples were fixed in FAA70 for 24 h. Subsequently, the plant material was pre-washed with phosphate buffer (0.1 M, pH 7.2), dehydrated using an increasing ethanol series (30% to 100%), pre-infiltrated, and infiltrated with historesin (Leica, Germany) following the manufacturer’s recommendations. The samples were then sectioned transversely at a thickness of 5 μm using a rotary microtome (Model 1508R, Logen Scientific, China) and stained with toluidine blue—a polychromatic staining method (0.05% phosphate buffer, 0.1 M, pH 6.8). This staining was used to evaluate and quantify the occurrence of tissue necrosis and plasmolysis. Images were captured using an Olympus microscope (BX61, Tokyo, Japan) coupled to a DP-72 camera using the bright-field option. Anatomical observations of both the epidermal faces and chlorophyll parenchyma were conducted.

### 4.4. Gas Exchange and Chlorophyll a Fluorescence

Gas exchange measurements were taken on the same day as the leaf measurements, and chlorophyll fluorescence was evaluated. Readings were conducted on fully expanded, unshaded leaves located at the second node below the apical meristem. An infrared gas analyzer (IRGA) model LI-6800 (Li-cor, Nebraska, USA) equipped with a blue/red light source was used. Net carbon assimilation rate (*A*, μmol m^−2^s^−1^), stomatal conductance (*gs*, mol H_2_O m^−2^ s^−1^), transpiration rate (*E*, mmol H_2_O m^−2^ s^−1^), internal CO_2_ concentration (*Ci*, μmol mol^−1^), and the ratio of internal to external CO_2_ concentration (*Ci*/*Ca*) were determined on fully expanded leaves in an open system under saturating light (1000 μmol m^−2^ s^−1^) and 400 μmol CO_2_ mol^−1^ air. The evaluations were performed between 8:00 a.m. and 11:00 a.m. under constant photosynthetically active radiation (PAR) of 1000 μmol photons m^−2^ s^−1^, CO_2_ concentration of ~415 μmol mol^−1^, temperature of ~25.5 °C, and relative humidity of ~74%, following the methodology proposed by Maxwell and Johnson [71].

The OJIP transient fluorescence of chlorophyll *a* was measured using a FluorPen FP 100 portable fluorometer (Photon Systems Instruments, Drasov, Czech Republic). Analyses were performed on the third leaf of all sample units. The leaves were dark-adapted for 30 min to oxidize the photosynthetic electron transport system completely. Subsequently, a pulse of 3000 µmol m^−2^ s^−1^ of blue light was offered, measuring the minimum fluorescence (F_0_) at 50 μs when all photosystem II (PSII) reaction centers were open, defined as step O, followed by step J (at 2 ms), step I (at 30 ms), and maximum fluorescence (F_M_) when all PSII reaction centers were closed, defined as step P. The values obtained for the different steps were used to estimate several bioenergetic indices of PSII, according to Strasser et al. [72], the specific light absorption flux per reaction center (ABSRC), specific dissipated energy flux at the level of the chlorophyll antenna complex (DioRC), photosynthetic performance index (PiAbs), and energy dissipation yield (PHIDo). Readings were taken 60 days after the application of sublethal doses of glyphosate to fully expanded leaves from the middle third of the plants.

### 4.5. Growth Analysis

After glyphosate treatment, biometric measurements were performed after 160 d of cultivation, including the height of *T. ciliata* plants (cm) and stem diameter (mm). Measurements were taken using a millimeter ruler and digital caliper, following Delarmelina et al. [73], to observe the morphological behavior over the months of plant development, according to the methodology proposed by Portes et al. [74].

### 4.6. Statistical Analysis

The obtained data were subjected to an analysis of variance (ANOVA) using the F-test and adjusted to regression models, adopting a significance level of 5%. The models were selected based on their simplicity, biological significance, and coefficient of determination. Subsequently, all variables that showed significant differences were jointly evaluated in a correlation matrix and associated using principal component analysis (PCA). Because these variables had different units of measurement, correlation PCA was performed using standardized data to obtain a mean of 0 and a standard deviation of 1. The number of principal components was defined according to the eigenvalues (>1.0) and explained variance (>70%). Statistical analysis was performed using R version 4.0.4 [75].

A matrix of similarities was compiled to determine the similarities or differences among plants subjected to different sublethal doses of glyphosate. The similarity index was obtained using the Pearson correlation coefficient, with *r* values transformed by d = (1 − r) × 100 to estimate the distance (d) values. The dendrogram was then generated using the Unweighted Pair Group Method with arithmetic averages (UPGMA), with adjustment between the distance matrix and the dendrogram estimated using a cophenetic correlation coefficient [76]. This analysis used the DendroUPGMA software (http://genomes.urv.es/UPGMA/) (accessed on 11 August 2022) [77].

## 5. Conclusions

Sublethal doses of glyphosate above 19.2 g ha^−1^ induced toxicity symptoms in *T. ciliata* leaves, ranging from mild symptoms, such as chlorosis, to severe symptoms, such as tissue necrosis. We observed a positive relationship between plant height gain and high photochemical yields with plant exposure to sub-doses 9.6 and 19.2 g ha^−1^. A sublethal dose of 38.4 g ha^−1^ improved the photosynthetic rate and carboxylation efficiency. The stem diameter of *T. ciliata* responded positively to increasing glyphosate doses. This occurs to compensate for the negative effect of glyphosate on water absorption. In this way, we confirmed the hypothesis of a hormetic effect on the exposure of *T. ciliata* to sub-doses of glyphosate equal to or lower than 38.4 g ha^−1^. However, the sublethal dose of 76.8 g ha^−1^ must be considered toxic, impacting the photosynthetic activity and, consequently, the height gain of *T. ciliata*.

## Data Availability

All the data relevant to this manuscript are available on request from the corresponding author.
